# The clinical significance of CDK1 expression in oral squamous cell carcinoma

**DOI:** 10.4317/medoral.19841

**Published:** 2014-08-17

**Authors:** Xin Chen, Feng-He Zhang, Qiao-Er Chen, Yuan-Yin Wang, Yin-Long Wang, Jia-Cai He, Jian Zhou

**Affiliations:** 1 Ph.D candidate, Department of Oral and Maxillofacial Surgery, College and Hospital of Stomatology (Shandong Provincial Key Laboratory of Oral Biomedicine), Shandong University, China. Lecturer, Department of Oral and Maxillofacial Surgery, College and Hospital of Stomatology (Oral clinical medicine key discipline of Anhui province, oral disease research provincial laboratory of Anhui province, the central and local governments construction center laboratory of stomatology), Anhui Medical University, China; 2 Professor, Department of Oral and Maxillofacial Surgery,College and Hospital of Stomatology, Shandong University, Shandong provincial key laboratory of oral biomedicine, China; 3 Professor, Department of Pathology, College and Hospital of Stomatology, Anhui Medical University, China; 4 Professor, Department of Oral and Maxillofacial Surgery, College and Hospital of Stomatology, Anhui Medical University, China

## Abstract

Objectives: To evaluate the clinical significance of cyclin-dependent kinase 1 (CDK1) in 77 oral squamous cell carcinomas (OSCC) using immunohistochemical methods. Study Design: Immunohistochemical expression of CDK1 was compared with various clinicopathological features in 77 OSCC and 60 controlled epithelia adjacent to the tumours. In addition, correlation of CDK1 expression and prognostic and the 5-year accumulative survival rate of OSCC were investigated. Results: The CDK1 protein was expressed in 52 cases of 77 tumor tissues (67.5%), compared with 21 cases of 60 controlled (35.0%). The expression of CDK1 was significantly correlated with the histological grade of OSCC (*P*<0.05). The CDK1 protein was over-expressed in recurrent tumors or in those with lymph node metastasis. Statistical analysis showed a significant reduction in the 5-year accumulative survival rate in CDK1 positive cases compared with CDK1 negative cases (*P*<0.05). Namely, the CDK1 positive patients had poor prognosis. Conclusions: The expression of CDK1 might serve as malignant degree and prognostic markers for the survival of OSCC.

** Key words:**Cyclin-dependent kinase 1 (CDK1), oral squamous cell carcinoma (OSCC), immunohistochemistry, cell proliferation.

## Introduction

Oral squamous cell carcinoma (OSCC) is the most common type of malignancy of the oral cavity (1) and the sixth most common type of cancer in the world. Each year more than 31,000 patients are newly diagnosed with OSCC in the USA (2). In China, the risk of morbidity due to OSCC is increasing, and the patients tend to be increasingly younger (3).

The cyclin-dependent kinases (CDKs) are master regulators of the cell cycle and are likely to play central roles in growth control during the cell cycle (4). CDK1 is a key factor for G2-M phase transition as well as cyclin B1 complex pushes cell from G2 phase to M phase and hence this is well-known as maturation promoting factor (MPF) (5). This complex performs chromatin condensation, nuclear envelope breakdown, fragmentation of golgi apparatus and endoplasmic reticulum as well as spindle formation by microtubule instability. Subsequently at prophase and at beginning of anaphase an ubiquitin ligase (E3) known as the anaphase-promoting complex/cyclosome (APC/C) will get attached to CDK1 and cyclin B1 complex which triggers the destruction of the mitotic cyclins (6). Dysregulation of the cell cycle machinery is a fundamental hallmark of cancer progression and the cell programmers of proliferation, differentiation, senescence and apoptosis are intimately linked to the cell cycle regulatory machinery. It has been clarified that the failure of cell proliferative mechanism is one of the malignant transformation factors (7-9). The highly conserved protein kinase CDK1 is a key component in the regulation of entry into mitosis in many eukaryotic systems. Regarding CDK1 as an index of cell proliferation is conceivable.

In this study, we examined the expression of CDK1 in 77 cases of OSCC using immunohistochemistry, and evaluated the clinical significance of its expression comparing to clinicopathological factors and prognosis.

## Material and Methods

-Patients and specimens

Seventy-seven patients diagnosed with primary OSCC were selected, including tongue cancer (n=26), buccal carcinoma (n=23), carcinoma of hard palate (n=8), upper or lower gingival carcinoma (n=17), floor of mouth carcinoma (n=3), who underwent a radical surgery procedure between December 1999 to June 2006 at Department of Oral and Maxillofacial Surgery, Hospital of Stomatology, Anhui Medical University, China. Clinical tumor stages were classified based on TNM criteria (UICC, 2010) (10). There were 14 cases in stage I, 27 in stage II, 23 in stage III, and 13 in stage IV. Hematoxylin and eosin stained slides from each biopsy specimen were reviewed, and histological grades were assigned in each case. Tumors were classified as grade I (well differentiated tumor), grade II (moderately differentiated tumor), and grade III (poorly differentiated tumor) tumor according to the WHO (2005) criteria (11). All patients underwent follow-up survey until death or for an average of 7.4 years (0.8 year~11. 7 years). In addition, 60 specimens of epithelia adjacent to the tumours from the same sample of OSCC were chosed as control group.

-Immunohistochemistry

Immunohistochemical staining for CDK1 was performed with a peroxidase-labeled streptavidinbiotin method on formalin fixed, paraffin embedded 4-micron sections from biopsy specimens in each case. The antigenic determinant sites were unmasked with 0.01 M sodium citrate solution in association with microwave irradiation. The sections were incubated at room temperature for 2 h with mouse IgG monoclonal antibody to human CDK1 fusion protein (1:50 dilution, Fuzhou Maixin Biotechnology Development Co., Ltd.kChina), and were subsequently incubated with a linking biotinylated anti mouse antibody (Fuzhou Maixin Biotechnology Development Co., Ltd. China) and a streptavidin-peroxidase label (Fuzhou Maixin Biotechnology Development Co., Ltd.China). After the development of the color with Diaminobenzidene substrate, the slides were counter stained lightly with hematoxylin. The sections of human tonsil were used as positive controls for CDK1 staining. Negative controls were the test tissue omitting the primary antibody from the incubation sequence. The cell stained nucleus or cytoplasm dark brown was regarded as CDK1 positive case. We counted the CDK1 positive cell in 1000 cancer cells, and evaluated as CDK1 positive case when its ratio was 10% or more (12).

-Statistical analysis

Data analysis was performed with SPSS 11.0 statistical packages. The differences in immunostaining and protein levels among each group were analyzed by Chi-square tests. Two-tailed Pearson statistics were used for correlated expression of these markers after confirmation of the sample with Gaussian distribution. Survivor functions were estimated by the Kaplan-Meier method, and survival curves were compared by the Log rank test (13). Statistical significance was defined as the *P*-value was <0.05.

## Results

-Expression and location of CDK1 in oral epithelia adjacent to the tumours and OSCC 

**Figure 1:**
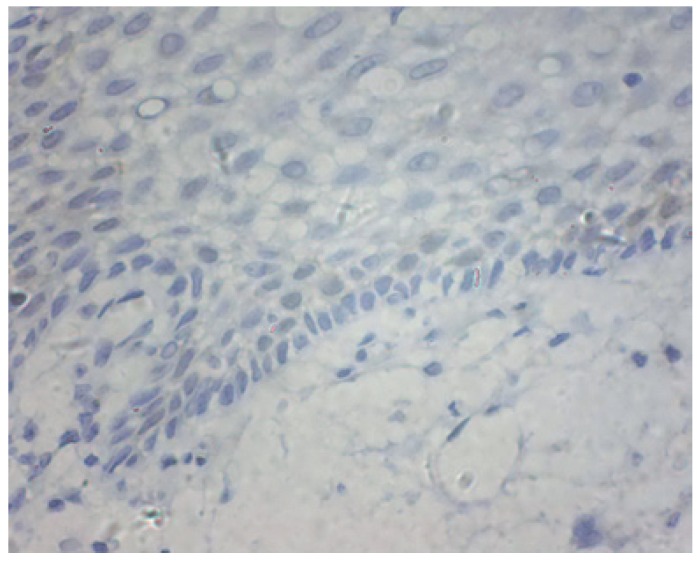
CDK1 protein locations were not found in epithelia adjacent to the tumours (original magnification:×400).

**Figure 2:**
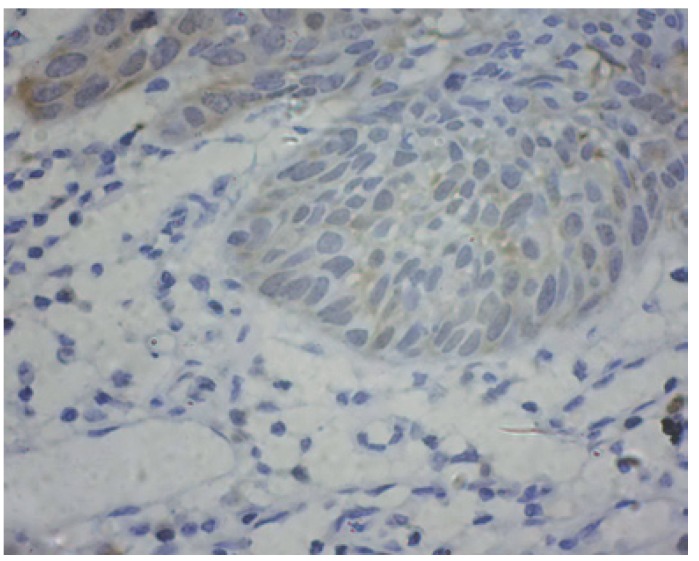
CDK1 protein was expressed in well differentiated squamous cell carcinoma (original magnification: ×400).

**Figure 3:**
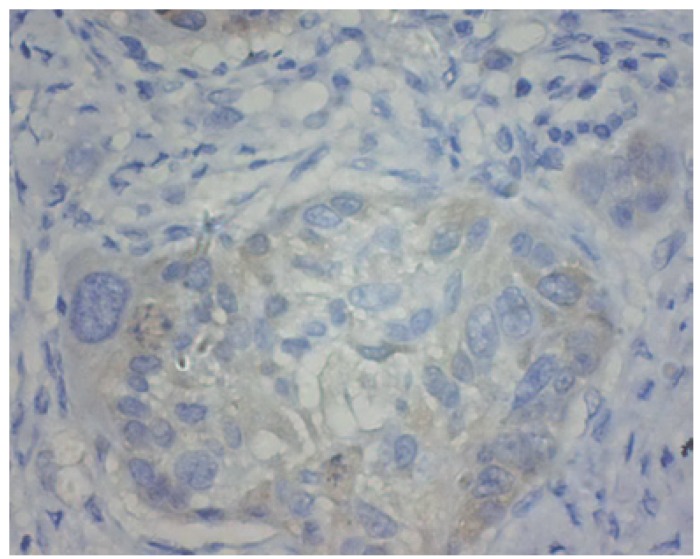
CDK1 protein was expressed in moderately differentiated squamous cell carcinoma (original magnification: ×400).

**Figure 4:**
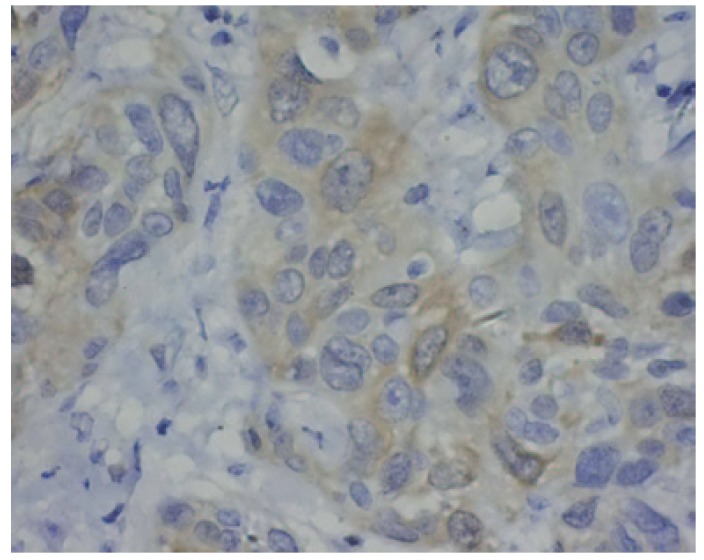
CDK1 protein was expressed in poorly differentiated squamous cell carcinoma (original magnification: ×400).

There was a significant difference in CDK1 expression between epithelia adjacent to the tumours and OSCC ( Table 1). CDK1 protein locations were not found in epithelia adjacent to the tumour cases (Fig. 1). As illustrated in figure 2,3,4, CDK1 protein was appeared in cytoplasm of the majority of OSCC cases. The scattered CDK1 positive cells were seen in diffuse invasive OSCCs (Fig. 4).

**Table 1:**
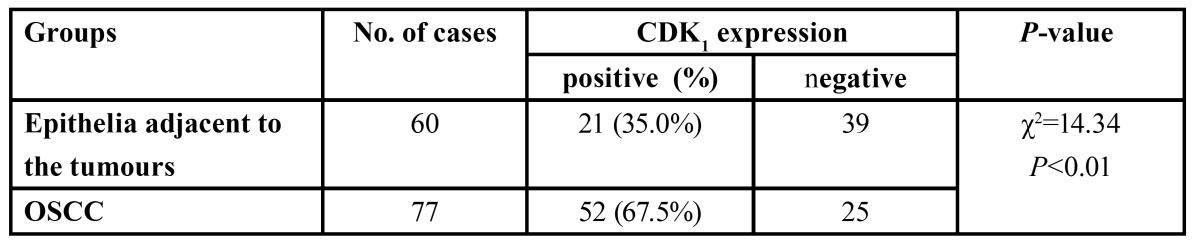
CDK1 expression between epithelia adjacent to the tumours and OSCC.

-Correlations between CDK1 expression and clinicopathological features of OSCC

The correlations between CDK1 expression and clinicopathological features were shown in  Table 2. There were no statistical differences in the expression of CDK1 protein and the clinicopathological features by age, gender and tumor location. The incidence of CDK1 protein expression increased gradually from stage I to stage III and from grade I to grade III, but decreased in stage IV. Differences in CDK1 protein expression among different clinical stages and histrogical grades were of statistical significance.

**Table 2:**
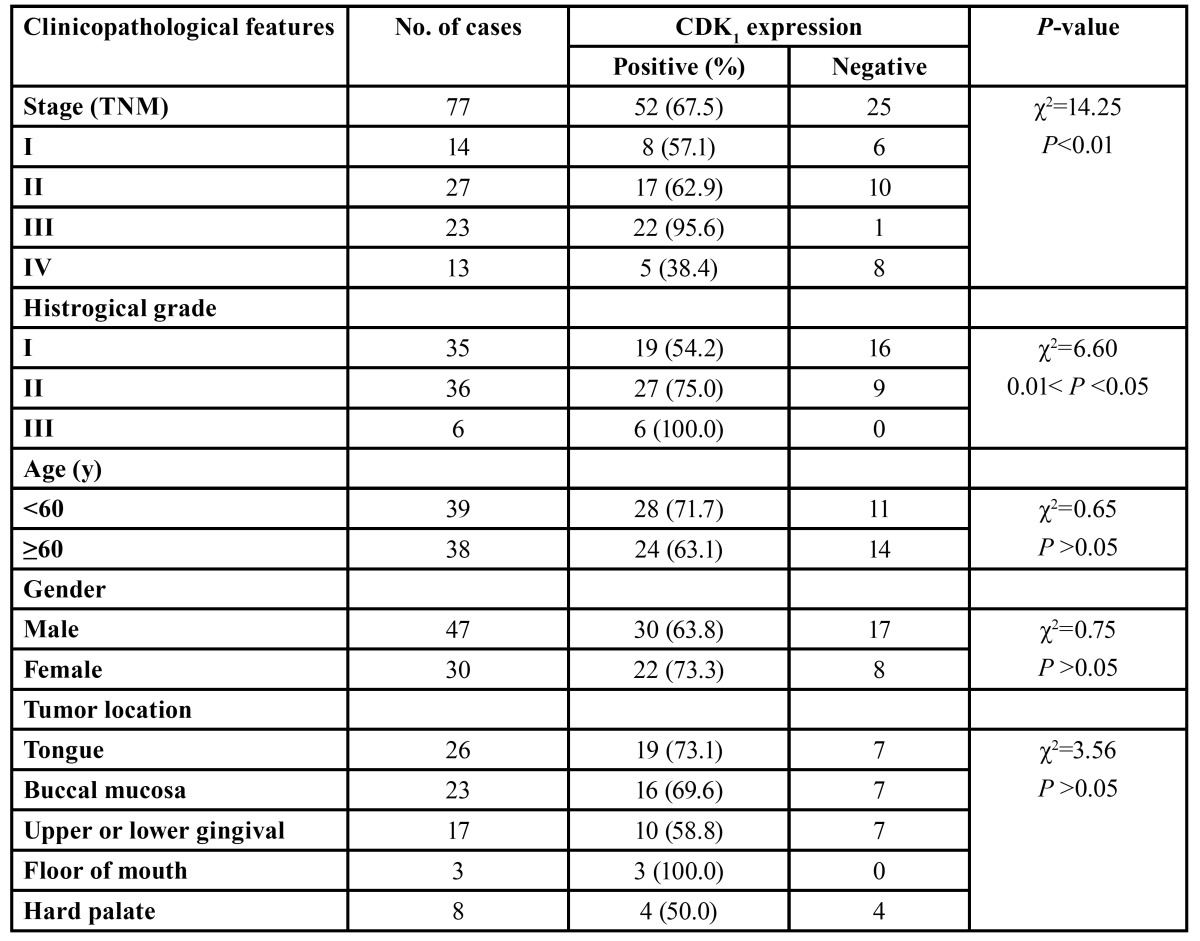
Correlation between CDK1 expression and clinicopathological features.

-Relationship between CDK1 expression and prognostic variables of OSCC

**Figure 5:**
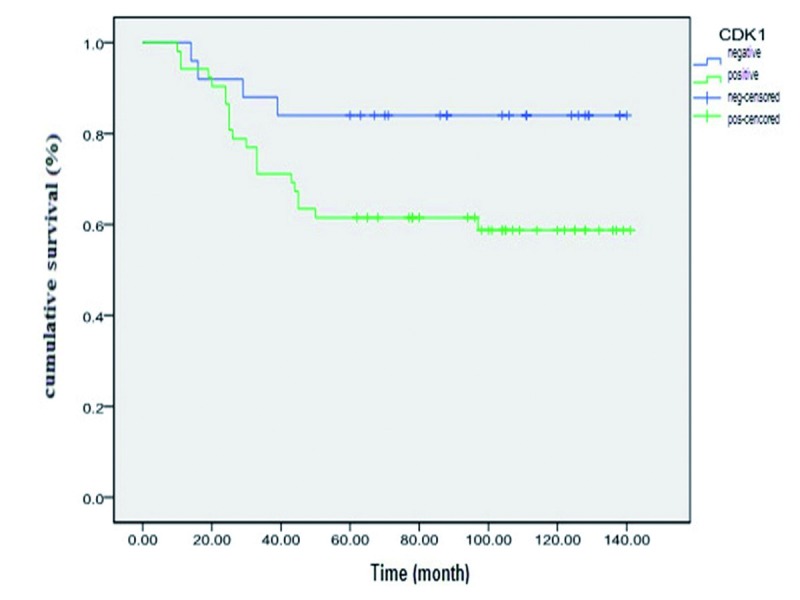
Survival curve of oral squamous cell carcinoma (OSCC).

CDK1 expression correlated with the occurrence of the tumor local recurrence, and the regional lymph node metastasis ( Table 3). In addition, the 5-year accumulative survival rate in CDK1 positive group was significant lower than that of the CDK1 negative group ( Table 4). The tumor prognosis in CDK1 positive group was obviously worse than that of CDK1 negative group (Fig. 5).

**Table 3:**
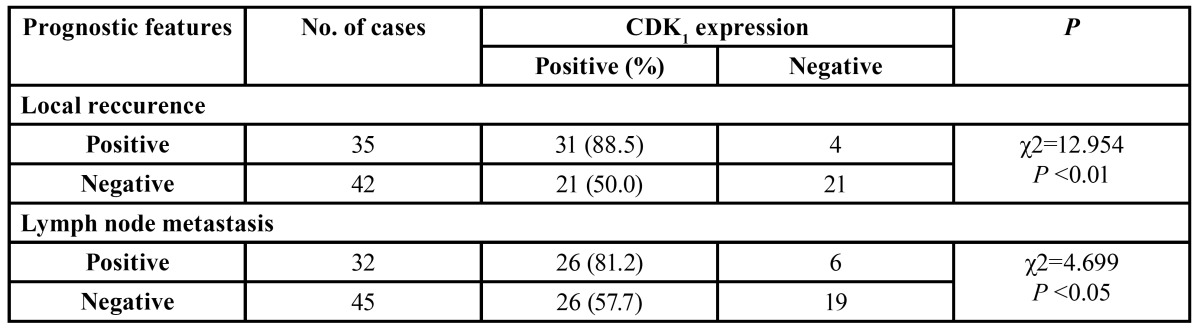
Relations of CDK1 positive and prognostic features OSCC.

**Table 4:**
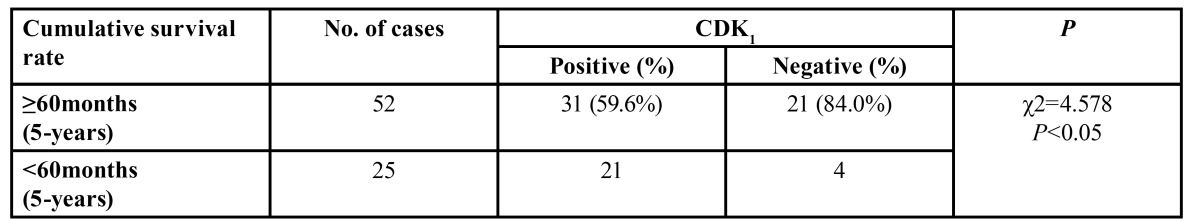
Relations Between CDK1 positive and 5-year accumulative survival rate.

## Discussion

Oral cancer comprises cancer of tongue, buccal mucosa, upper or lower gingival, floor of mouth and hard palate. More than 90% of cases of oral cancer are squamous cell carcinomas (14), which occurs at a relatively high frequency in China. OSCC is a serious and growing problem in many parts of the globe, mainly due to its low survival rate and poor life quality, especially for advanced cases (15).

To date, the most effective treatment for OSCC is extensive surgical resection, with radiotherapy and/or chemotherapy prior to or following surgery (16,17). Despite substantial effort and novel therapeutic developments, the five year survival rate for OSCC has not appreciably improved over the last two decades (18,19). However, patients are frequently diagnosed at late stages. Their different clinicopathological features, such as TNM stage, histological grade and tumor invasion, could reflect their clinical malignancy and clinical prognosis. Therefore, malignancy of OSCC was generally evaluated according to the clinicopathologic morphological features of the tumor synthetically, and chosen proper therapeutic program. However, it is not rare that clinical progression is inconsistent with degree of malignancy, thus the authors proposed that there might be complicated relations with other cytobiologic malignant factors, including the cell proliferation which could not been reflected by morphological features (12). The present study attempted to discover suitable novel biomarkers which reflect the physiological state and changes of cells prior to or during the disease process to offer early and accurate prediction and diagnosis for patients with OSCC, particularly in early stage OSCC (20).

In recent years, researches on cell proliferation associated proteins have been making considerable progress. CDK1 which located in the downstream of signal transcription during mitosis reflects the ability of cell proliferation directly. CDK1-mediated control of cell cycle might correlate with accurate reproduction and cell size homeostasis (21).

In this study, we performed an observation on CDK1 oncogenic properties, on the relationship between CDK1 expression and clinicopathologic parameters in OSCC. In the results of our study, CDK1 protein was expressed in 52 of 77 OSCCs (67.5%), as compared with 21 of 60 controlled epithelia adjacent to the tumours (35.0%, *P*<0.01). Concerning the intracellular locations of CDK1 protein in this study, the majority of CDK1 protein was present in cytoplasm. Goodger *et al*.(22) reported that both cytoplasm and nucleus in interphase cells or majority cells were stained by CDK1 specific staining, and it was over-expressed strongly in higher malignant tumor. In our study, CDK1 expression did not always appeared on case with high malignant clinicopathologic findings. Therefore, it is necessary to investigate the meaning of CDK1 intracellular location by increasing cases. As for the relation to the clinical stage of OSCC, the incidence of CDK1 expression increases along with the progression of tumor stage until stage III, but it decreases in notable advanced tumor, stage IV. This phenomenon is hard to explain from biological view. However, from this result, we hypothesized that cell proliferation showed a slow process in early stage carcinoma, and this process accelerated along with the progression of tumor, but in the advanced phase of cancer, the self-proliferation of cancer cells also will decline together with reduction of immune function. Thus the proliferative capacity of cancer cells was not considered always stable and invariable, and it might be promoted or repressed along with some changes of patient’s body condition. The study also found that CDK1 protein was over-expressed in high malignant (grade III) cancer. It was considered that variation in normal cell proliferative might reflect the clinical findings and histological malignancy to a certain degree (12).

Our findings showed that CDK1 protein was over-expressed in postoperative recurrence cases or in those with lymph node metastasis. Moreover, five-year accumulative survival rate was significantly higher in CDK1 negative cases than that in positive cases, both of which indicated that CDK1 protein might predict prognosis of OSCC. Wada *et al*. (12) reported the correlation between cdc2 (CDK1) expression and the prognosis of tongue SCC on their study, and described that the cdc2 may be an independently prognostic factor. These results reveal the physiological malignant degree of OSCC and suggest that the CDK1 might be a useful index for evaluating prognosis of OSCC. Further investigations were expected.

In conclusion, this study found the significant correlations between CDK1 expression and histological malignant grade, and survival rate of OSCC. These results indicated that the CDK1 expression might reflect the malignancy of OSCC, and it could be a useful index for determine malignant degree and the prediction of prognosis in OSCC.
